# Verbal Encoding Strategies in Visuo-Spatial Working Memory

**DOI:** 10.5334/joc.406

**Published:** 2025-01-06

**Authors:** Joana Pereira Seabra, Vivien Chopurian, Alessandra S. Souza, Thomas B. Christophel

**Affiliations:** 1 Department of Psychology, Humboldt-Universität zu Berlin, Berlin, DE; 2 Bernstein Center for Computational Neuroscience Berlin and Berlin Center for Advanced Neuroimaging, Charité Universitätsmedizin Berlin, corporate member of the Freie Universität Berlin, Humboldt-Universität zu Berlin, and Berlin Institute of Health, Berlin, DE; 3 Faculty of Psychology and Education Sciences, University of Porto, PT

**Keywords:** categorization, working memory, verbal labeling, visuo-spatial stimuli

## Abstract

Visual working memory and verbal storage are often investigated independently of one another. However, a growing body of evidence suggests that naming visual stimuli can provide an advantage in performance during visual working memory tasks. On the other hand, there is also evidence that labeling could lead to biases in recall. Here, we present an exploratory investigation of verbal labels associated with the memorization of simple visuo-spatial stimuli, and how the use of these labels informs recall behavior of the same stimuli in a separate working memory task. English-speaking participants performed a working memory task with orientation and location stimuli, followed by a separate naming task featuring the same stimuli. We found a diverse set of labels employed frequently and with a consistent distribution across stimulus types, the stimulus space, and among participants. The use of individual spatial words, predicted class 1 cardinal biases in memory (i.e. the observation that cardinal stimuli are more accurately recalled than non-cardinal ones). Conversely, words expressing uncertainty (e.g. ‘slightly’, ‘near’) predicted class 2 cardinal bias (i.e. recall biases away from the cardinal planes). This relationship between word use and recall biases is consistent with shared representational resources that are used for both visuo-spatial and verbal working memory.

## 1. Introduction

People often name visual stimuli to memorize them more easily. This labeling behavior divides stimuli into different categories and changes how we recall the original stimulus (Bae et al., 2015; Gauthier et al., 2003; Lupyan et al., 2007; Overkott & Souza, 2023; Souza et al., 2021). While one might think of it as a rare, ‘alternative’ strategy, verbal labeling may be an integral part of how we remember visual stimuli (Baddeley, 2003; Baddeley & Hitch, 1974; Forsberg et al., 2020; Glanzer & Clark, 1964; Grill-Spector & Kanwisher, 2005; Maier & Abdel Rahman, 2018; Yan et al., 2021). Understanding verbal labeling might help us understand how we use mixed-modality benefits to increase precision (Brown & Wesley, 2013; Chopurian et al., 2024; Cohen et al., 2014), how we prioritize individual items (Christophel et al., 2017; Emrich et al., 2017), and how we compress large quantities of visual information using chunking (Chen & Cowan, 2009; Thalmann et al., 2019). However, how we decide what labels to use for a given stimulus and how this decision affects recall behavior is poorly understood.

Naming or describing an item when memorizing visual stimuli has been understood as a common occurrence since early in working memory research (Baddeley, 2003; Baddeley & Hitch, 1974; Glanzer & Clark, 1964). Labeling stimuli has proven to improve accuracy in some visual working memory tasks (Forsberg et al., 2020; Gauthier et al., 2003; Lupyan et al., 2007; Overkott & Souza, 2023; Souza et al., 2021), and increasing specificity in the verbal description for colors and shapes has been shown to lead to even better performance (Hasantash & Afraz, 2020; Souza et al., 2021). Increased complexity in abstract stimuli tends to elicit more complex verbal labels, and stimuli that are difficult to label are also linked to lower working memory accuracy (Brown et al., 2006; Orme et al., 2017). On the other hand, verbalization of visual stimuli can also induce biases and reduce recall accuracy (Brandimonte et al., 1997; Schooler & Engstler-Schooler, 1990). Furthemore, attributing the same broad category to several visual stimuli can create confusion when recalling and distinguishing between them (Lupyan, 2008). We also often classify and label visual stimuli without prior intention or instruction (Baddeley, 2003; Baddeley & Hitch, 1974; Grill-Spector & Kanwisher, 2005; Maier & Abdel Rahman, 2018; Yan et al., 2021). Consistently, articulatory suppression leads to worse working memory performance when compared to a labeling condition (Overkott & Souza, 2023; Souza et al., 2021) and impairs language learning (Papagno et al., 1991). These findings seem to make the existence of a close relationship between visual and verbal working memory evident, but the interaction between the two is not yet completely clear.

On the neural level, both visual and verbal working memory have been extensively investigated, and both seem to depend on distributed cortical representations (Buchsbaum & D’Esposito, 2019; Buchsbaum & Esposito, 2008; Christophel et al., 2017; Emch et al., 2019; Eriksson et al., 2015; Lee & Baker, 2016). Visual working memory in its purest form is most commonly found to rely on low-level sensory representations in sensory cortices (Awh & Jonides, 2001; Harrison & Tong, 2009; Postle, 2006; Serences et al., 2009). Regarding verbal working memory, regions that are known to be essential for the perception and production of speech also show increased activity during verbal working memory when compared to non-verbal tasks or across load-conditions (Yue et al., 2019). Patients with damage to some of these regions exhibit impairment not only in tests of language comprehension and production but also in verbal working memory tasks (Koenigs et al., 2011; Warrington & Shallice, 1972). Recent multivariate decoding work shows that information about individual memorized characters can be found in anterior parts of this language network (Yan et al., 2021). But working memory representations do not solely rely on primary sensory and language-related regions; instead, they seem to form a continuum from sensory to abstract representations (Christophel et al., 2017; Fuster, 1997). Consistently, representations of semantic meaning reach far beyond the limits of language networks, including sensory regions (Deniz et al., 2019; Hebart et al., 2023; Mitchell et al., 2008; Popham et al., 2021). Conversely, visual representations extend beyond visual cortices (Christophel et al., 2012; Ester et al., 2015) and appear to be represented using neural codes that are abstracted from the originally encoded stimulus even in sensory cortices (Kwak & Curtis, 2022). Recent work also provides evidence for verbal representations during visual working memory by showing that color stimuli in the extrastriate visual cortex are retained using a categorical code instead of a sensory one (Yan et al., 2023). Crucially, this categorical code has been identified using a verbal encoding model based on participants’ labeling data for the colors used in the study. This suggests participants mostly relied on a more abstract type of representation to retain these color stimuli, and not a veridical, fine-grained one. Generalization between different types of visual stimuli also provides support for this use of abstract representations during visual working memory (Pereira Seabra et al., 2023). This neural evidence suggests that abstract representations (including verbal ones) are frequently present during visual working memory, consequently playing a role in the categorization of these stimuli.

On the behavioral side, categorization of visual stimuli can improve recall accuracy, but can also lead to categorical biases. Categorical biases can induce uncertainty during stimulus recall and benefit some stimuli to the detriment of others. Prototypical stimuli of a given category are often remembered better than the ones at the edges of two categories (Bae et al., 2015; Pratte et al., 2017). This is probably because stimuli at the edges of two categories can induce confusion in their naming. For instance, when shown an ultramarine colored stimulus, most people will confidently refer to it as ‘blue’, but will likely hesitate when deciding if turquoise falls under ‘blue’ or ‘green’. Categorical biases have not only been reported for color stimuli but also for visuo-spatial stimuli, both during visual (Bae, 2020; Balikou et al., 2015; Essock, 1980; Girshick et al., 2011; Huttenlocher et al., 2004) and haptic tasks (Baud-Bovy & Gentaz, 2012; Essock et al., 1992). In visuo-spatial working memory, recall performance is subject to cardinal biases, as indicated by subjects (a) reproducing horizontal or vertical (i.e. ‘cardinal’) lines more accurately then diagonal ones (visual, class 1 biases) and (b) recalling diagonal stimuli as if they were shown further away from the cardinals than their original position (cognitive, class 2 biases; Balikou et al., 2015). It is unclear, however, whether these biases directly map onto labeling behavior during memory encoding and whether word use can predict the extent of recall bias.

Here, we aimed to explore how subjects label simple, low-level visuo-spatial stimuli and to relate their labeling behavior to their recall performance. Specifically, we asked whether labeling could predict and explain why a particular stimulus will be subject to cardinal biases and why another stimulus will not. To investigate this, we collected data from subjects who performed orientation and location delayed estimation tasks. Afterwards, in a separate task, the same subjects were asked to freely label location and orientation stimuli. We firstly map out how subjects label simple, low-level visuo-spatial stimuli across the respective feature spaces. We then examined whether these labels from the verbal task and their use are predictive of behavioral performance and biases in the delayed estimation task.

## 2. Methods

### 2.1 Participants

One-hundred thirty-three participants were recruited using Prolific and received monetary compensation of £8,50 per hour. The final sample size was not defined a priori. We initially collected data from 31 participants, and later decided to increase the sample size in order to obtain stable estimates of memory performance and labeling behavior, collecting data from an additional 102 subjects. We aimed for a high number of participants within the range of other recent behavioral verbal labeling studies (Hasantash & Afraz, 2020; Overkott et al., 2023; Overkott & Souza, 2022, 2023) because we wanted to create a robust dataset that could be shared and used in future research. Subjects were all between 18 and 45 years old (*M* = 32, *SEM* ± 0.57). All participants reported to be fluent in English. Participants from the second data collection (N = 102) self-identified as monolingual native-English speakers, while no clear native language information was recorded for the subjects in the initial data collection (N = 31). Participants gave informed consent. The study was approved by the internal review board of the Institute for Psychology of the Humboldt-Universität zu Berlin (IRB number: 2021–08). We excluded a total of 25 participants (2 from the first batch of data collection, 23 from the second) due to poor data quality: 3 due to average error superior to 45 degrees, 5 due to not adjusting the random starting value during recall in over 8% of the trials, 2 due to not logging a response for over 15% of the trials, 7 due to not following task instructions, and 8 participants whose average recall error was more than 2 standard deviations away from the pool average were also removed. Data from the remaining 108 participants was analyzed.

### 2.2 Experiment

#### 2.2.1 Delayed Estimation Task

The background was gray (RGB: 128,128,128) throughout all tasks. The first part consisted of two delayed estimation tasks, one featuring oriented grating stimuli (hereafter orientations), and another featuring spatial location stimuli (hereafter locations). There were 24 orientation stimuli and 24 location stimuli, each set of stimuli covering the entire feature space in equidistant positions. However, the stimuli in each set had different angular positions, as the oriented gratings are a mirrored stimulus, only needing 180° (instead of 360°) to cover the entire feature space. Therefore, the 24 orientations were separated from each other by 7.5 degrees, starting at 0° (vertical), and ending at 172.5°. Location stimuli had a distance of 15° between one another, also starting at 0° (top) and ending at 345°. There was a smooth fixation dot in the center of the screen for both tasks. Both stimuli were confined to a 651 × 651 pixel invisible square in the center of the screen. The orientation stimulus was a black and white Gabor patch with blurred edges (gaussian kernel = 1°, sd = 0.5°, spatial frequency = 60 pixels/cycle) and an annulus for the fixation dot (12px diameter). The location stimulus was a dot (31px diameter), larger than the fixation one, that was placed along an imaginary circle (235px radius) with the fixation dot in the center.

**Figure 1:**
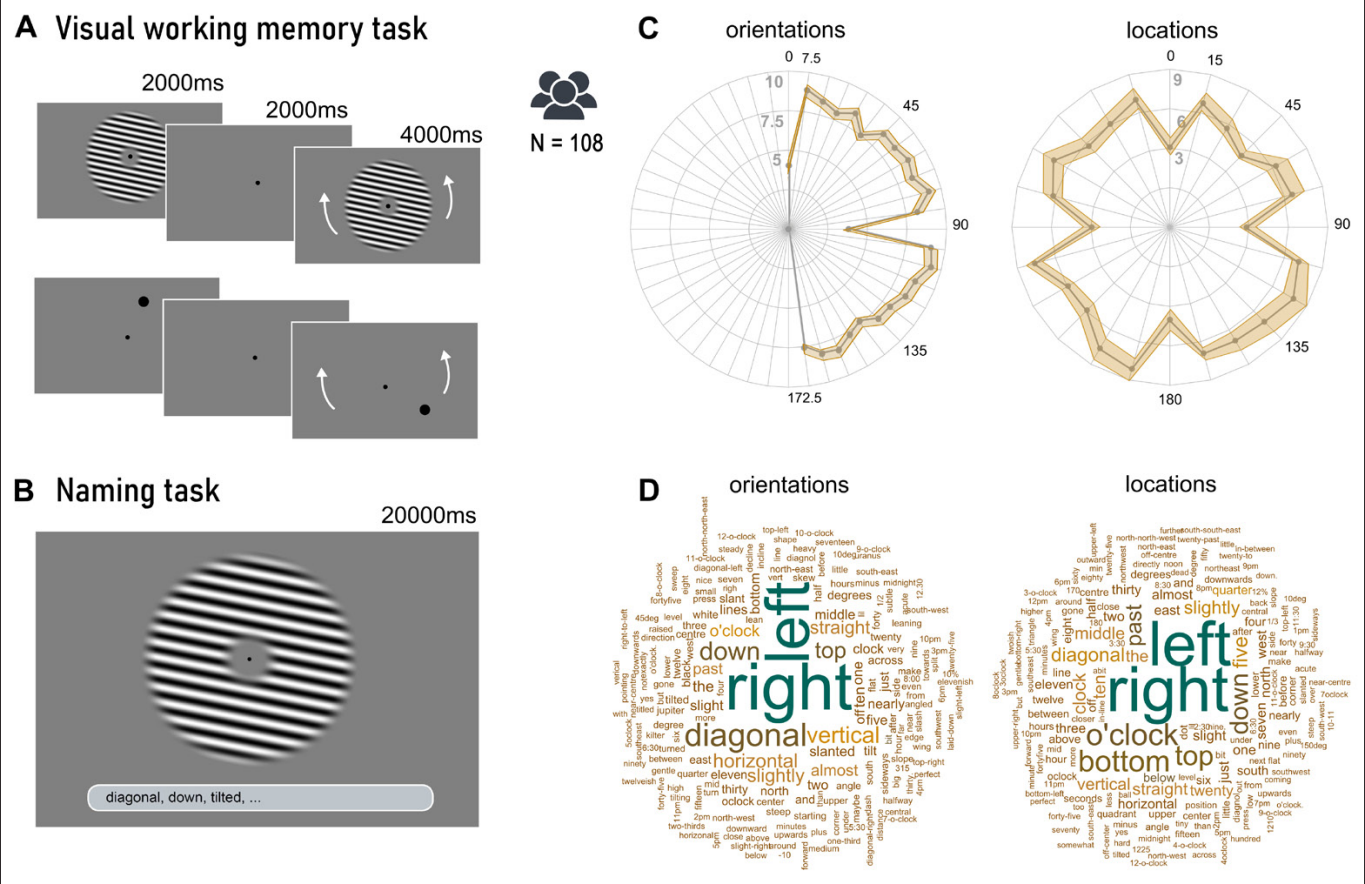
Delayed Estimation and Naming Tasks’ Design and Results. *Note.* Delayed Estimation Task, Naming Task, and respective results. (A) Visual working memory paradigm. This was a delayed estimation task where participants were required to memorize the rotation of an orientation or location stimulus and reproduce it after a short delay by rotating a probe. (B) Naming task paradigm. Participants viewed either an orientation or location and were asked to input words that would describe the stimuli and help them memorize that specific stimulus. (C) Recall error for each stimulus in orientation and location trials. Distance from the center to the edge of the plot represents the average error for each stimulus. (D) Word clouds of participants’ answers to the naming task. The size of each word depicted is representative of how often it was employed during the task by the entire participant pool.

Figure 1 A illustrates the flow of events in the delayed estimation tasks. In each trial, a single stimulus (either an orientation or a location) was show for 2000 ms. After a delay of 2000 ms, participants were asked to reproduce the orientation or location of the original stimulus. They were given 4000 ms to adjust the probe using the keys ‘X’ and ‘M’ on their keyboard and press the spacebar to log their response. When failing to adjust the probe within the given time frame and/or not logging their response by pressing the spacebar, there was a reminder after the trial was over that read ‘You didn’t press the spacebar. After rotating the image, please log your answer by pressing the spacebar’.

#### 2.2.2 Naming Task

Figure 1B illustrates the naming task. The naming task featured the same 24 orientation and 24 location stimuli as the delayed estimation task. A prompt read ‘Type words or combinations of words that describe the orientation of the image’ in the trials with orientation stimuli, and ‘Type words or combinations of words that describe the position of the big dot’ in the trials with locations. Underneath this prompt was an open field for participants to type their answers. Subjects were instructed to use commas to separate multiple entries. Participants were instructed to ‘write down words that you would use to memorize the orientation or location of the stimulus you are seeing’. Each trial lasted up to 20 seconds, subjects could press the spacebar to continue to the next trial, and there was a 500 ms inter-trial interval.

### 2.3 Procedure

Participants took part in the study on their own desktop or laptop computer. Due to the experiment taking place online, we could not control for retinal stimulus size. After providing informed consent, participants received instructions about the delayed estimation task and performed six training trials with trial-wise feedback. To ensure understanding of the task, we asked participants three multiple choice questions after the training trials. Participants received instructions about the naming task after finishing all delayed estimation task blocks, and prior to the two naming task blocks. They performed a demo of the task with 3 trials of orientation and 3 trials of location stimulus. Participants answered three task-relevant multiple choice questions after the demo.

The experiment consisted of a delayed estimation task, followed by a naming task. There were 10 blocks of the delayed estimation task (5 featuring orientations, another 5 featuring locations), each block lasting about 2.5 to 3 minutes. Afterwards, there were 2 blocks of the naming task (1 featuring orientations, 1 featuring locations) each block lasting about 3 minutes. For the delayed estimation task, blocks were grouped into pairs (one for each stimulus type – orientations and locations) and their order of appearance was randomized within each pair. The order of appearance of the two blocks of the naming task was also randomized.

Before starting the delayed estimation task, half of the participants received a pre-experimental prompt that read ‘During the delay between the two images consider what words might be helping you memorize the orientation or location of the stimulus. Later in the experiment we will ask you about the process you used to solve this task’. The other half did not receive this prompt. We included this manipulation to compare whether the behavior of participants who received an explicit request to verbalize the stimuli would differ from those who did not. All participants were reminded to press the spacebar in every trial to log their answers before the start of the experiment. Each of the delayed estimation and naming task blocks featured 24 trials. Stimuli were not repeated within a block, meaning that each block included every single one of the 24 stimuli exactly once, in random order.

After completing all the tasks, participants were asked to fill out a questionnaire (see Appendix A). It included ratings and open-field questions about their memorizing strategies during the delayed estimation task, and the possibility to provide feedback about the study. The study took around 50 minutes for each participant to complete. Participants were able to take 2-minute breaks 3 times throughout the experiment (roughly every 15 min), but they could choose to skip the break.

### 2.4 Data Analysis

We analyzed recall errors and naming behavior using R version 4.1.1 (R Core Team, 2021) and R Studio (RStudio Team, 2020). To assess cardinal biases, stimuli were divided into 2 categories: cardinal (0°, 90°, 180°, 270°) and non-cardinal (all other stimuli). Absolute recall error differences between the two categories were assessed using a one-sided paired t-test. Directional biases were tested by selecting stimuli close to the cardinals (up to 15° away in either direction) and far from the cardinals (22.5° to 67.5°) and assessing their relative recall error, i.e. value of recall error after correcting for error direction. A one-sided paired t-test was performed to compare the near-cardinal and far from cardinal groups. To compare recall error in orientation and location stimuli, a two-sided paired t-test was used. Performance of participants who received the pre-experimental prompt was tested against those who did not with a one-sided unpaired t-test.

We ran an across-participants Pearson correlation for each verbal strategy comparing mean absolute recall error in the delayed estimation task to total number of naming trials per participant in which that labeling strategy was used. The same correlation was used to assess the relation between recall error to total number of strategies used per participant. Across-stimuli Pearson correlations were employed for each strategy when assessing recall error in relation to total trials per stimulus in which the strategy was used. The same procedure was used when comparing recall error to each individual spatial language word use, when comparing directional biases to each approximation word’s use, and when comparing word use for orientations and locations.

Responses to the post-experimental questionnaire on encoding strategies were compared between orientations and locations. A Wilcoxon Signed-Rank test was used to assess the scores for each strategy. To test for high-labeling specificity benefits, we assessed non-cardinal stimuli’s recall error differences in two sets of analyses: (1) number of strategies used to describe a given stimulus per naming trial and (2) number of spatial language words used. We asked whether these measures of labeling specificity predicted recall error with Pearson correlations, both across-stimulus and across-participants. In the across-stimulus analysis, we assessed, for example, whether the average amount of strategies most frequently employed across all subjects for each stimulus correlated with its recall error averaged across subjects. For the across-participants test, we tested whether participants’ strategy use, averaged across trials, is predictive of their performance. We also used a Pearson correlation to test whether participants who used a higher number of unique terms during the naming task were more accurate in the delayed estimation task.

### 2.5 Glitches

For the first 30 participants, we had not yet included the restriction to English monolingual speakers. This sample includes native English-speakers (not necessarily monolingual) and native Portuguese, Hungarian, Polish, Spanish, Italian, Slovenian, Greek, Czech and Turkish speakers. After this initial data collection, we decided to restrict participation to native English speakers.

Initially, the experiment had an inactivity timeout; if participants did not use their keyboard for longer than 5 minutes, a message would appear on the screen asking if they were still taking part in the study and, after another 10 seconds of inactivity, they would be redirected out of the study. The purpose of the inactivity timeout was to prevent bad faith actors from being able to remain in the experiment while inactive and subsequently ask for monetary compensation, as well as saving us time during data quality checking. However, several participants reported the inactivity message appearing even when they were active, which we confirmed. The inactivity timeout was therefore removed after the first 21 participants.

## 3. Results

### 3.1 Cardinal biases during orientation and location memory

Recall error was significantly lower for cardinal stimuli (orientations: *M* = 3.90, *SEM* ± 0.24, locations: *M* = 4.65, *SEM* ± 0.25) when compared to non-cardinal stimuli (orientations: *M* = 8.35, *SEM* ± 0.08, locations: *M* = 7.24, *SEM* ± 0.13) (class 1 cardinal biases) for both orientations (see Figure 1C, *t*(107) = –13.253, *p* < .001, *d* = 1.57) and locations (see Figure 1C, *t*(104) = –11.833, *p* < .001, *d* = 1.1). We then checked whether these errors had a directional bias (class 2 biases). We found that orientations and locations near the cardinals (between –15° and +15°) showed significantly larger errors away from the cardinals then stimuli with a larger distance to the cardinals (22.5° to 67.5°) (orientations: *t*(105) = 10.283, *p* < .001, *d* = 1.04; locations: *t*(107) = 7.02, *p* < .001, *d* = 0.88). No significant difference was observed between absolute recall error in participants who received the pre-experimental verbal prompt and those who did not receive it for orientations (*t*(106) = –0.643, *p* = .521, *d* = 0.12) nor for locations (*t*(106) = 1.429, *p* = .156, *d* = 0.28).

### 3.2 Subjects mostly use spatial and clock-analogous terms

**Figure 2:**
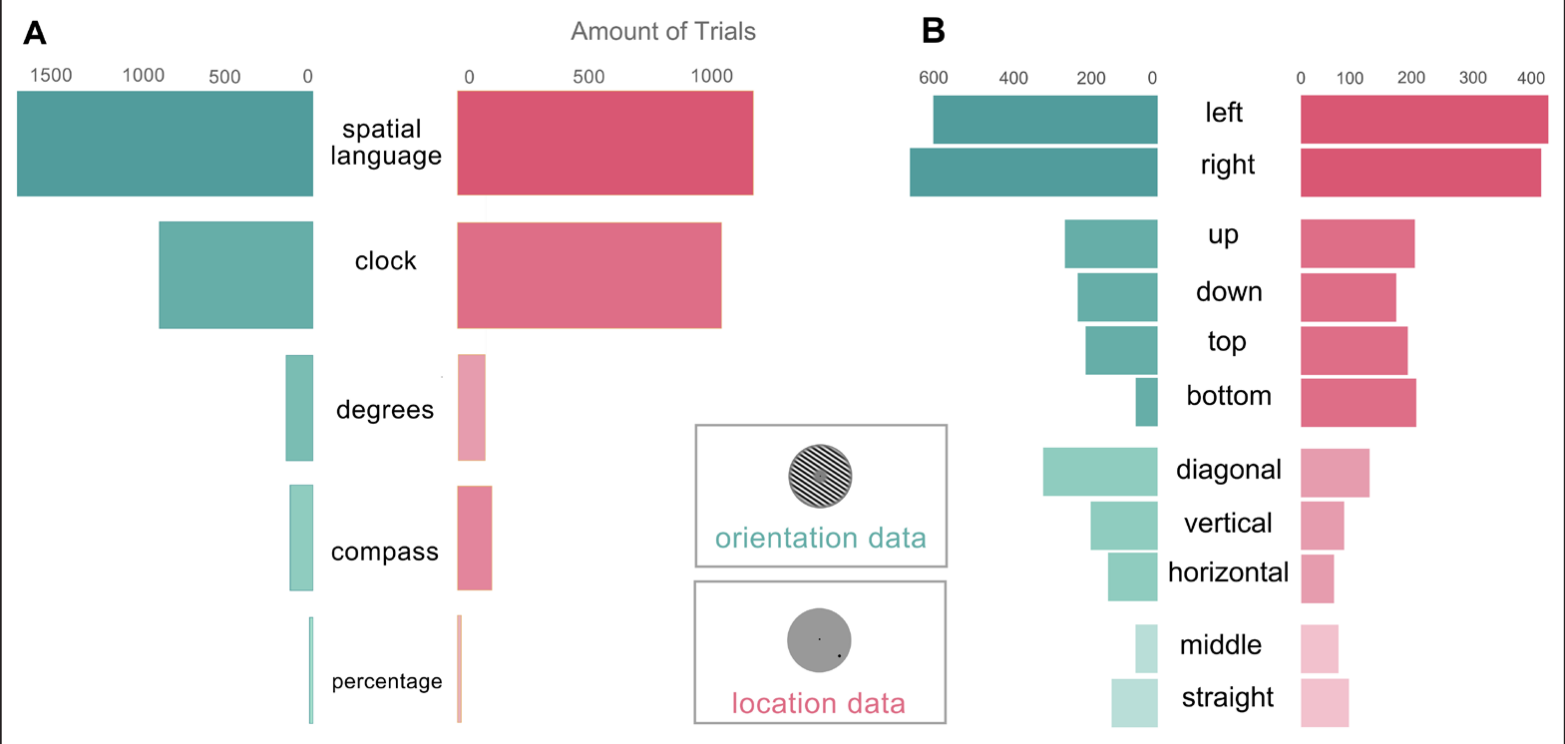
Main Labeling Strategies and Spatial Words Usage During the Naming Task. *Note.* Usage of the main strategies and spatial words in the naming task, in both orientation and location stimuli. ^A^ Frequency of Label Use in orientation (in green) and location (pink) trials. Amount of total naming task trials with orientation stimuli in the entire participant pool in which words associated with each of the main strategies were employed. ^B^ Frequency of Spatial Language terms used in orientation trials (left) and location trials (right).

In the naming task, we asked participants to input words ‘that you would use to memorize the orientation or location of the stimulus you are seeing’. Overall, subjects used about 1900 unique word entries (including typos and variations, see Figure 1D) across 8913 total entries. Many words were reported by several participants repeatedly. Post-hoc, we grouped words together into five categories: spatial language, clock analogies, navigational analogies, degrees of rotation and percentages or ratios (Figure 2A). After an initial inspection of the verbal data, we defined labeling strategies based on the more frequently used sets of words with a common conceptual origin. We manually classified each verbal entry with one or more labeling strategy. Words that appeared rarely or whose meaning was not clear were not included in this classification process.

Subjects frequently used spatial language like ‘right’ and ‘left’, ’up’ and ‘down’ (or a variant thereof, like ‘top’ and ‘bottom’), ‘diagonal’, ‘vertical’ and ‘horizontal’, ‘straight’, ‘middle’ (Figure 2D). These were the most used words, whether alone, in combination with one another, or in combination with other words. Participants also used clock-analogous labels (‘3 o’clock’), navigational compass-analogous terms (e.g. ‘north’), quantified locations and orientations in degrees, or described percentages and ratios relative to a standard angle. Along with these five main strategies, two groups of words were frequently used to describe stimuli: approximation words and precision words. Approximation words describe a stimulus’ relation to another stimulus or position – such as ‘slightly’, ‘almost’, ‘just’ and ‘near’. On the other hand, precision words reiterate a stimulus position – for instance, ‘exactly’, ‘dead on’ or ‘precisely’. We found no correlation between recall error and number of trials in which a participant used any of the five strategies (all *p* > .2) nor when assessing these correlations for each stimulus modality (all *p* > .2) and no correlation between task performance and number of main strategies used by each participant (all *p* > .05).

### 3.3 Spatial word usage predicts cardinal biases

**Figure 3:**
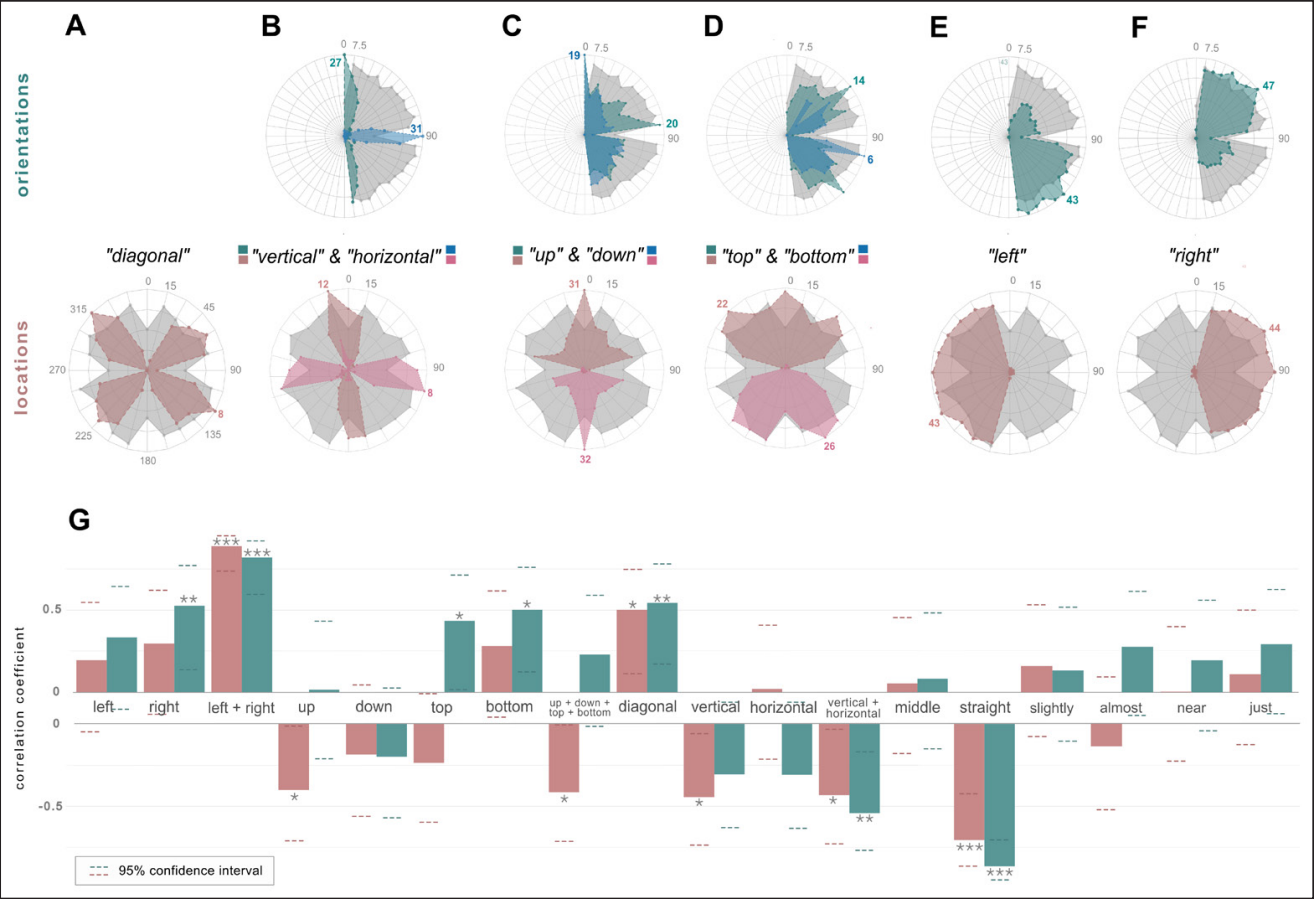
Spatial Language Words Usage During Naming Task and Correlation with Delayed Estimation Task Recall Error. *Note.* Distribution of usage of spatial language words across the stimulus space and their correlation to recall error in delayed estimation task. (A) Use of the word ‘diagonal’ in orientation and location trials. The area shaded in color corresponds to the amount of times the word was used for a given stimulus, and the number with matching color indicates (in the stimulus in which it was used more frequently) the number of participants who employed it to describe that specific stimulus. The gray area indicates the average absolute recall error for each orientation stimulus. (B) Use of the words ‘vertical’ (green for orientations, dark pink for locations) and ‘horizontal’ (blue for orientations, light pink for locations) in orientation and location trials. (C) Use of the words ‘up’ (green for orientations, dark pink for locations) and ‘down’ (blue for orientations, light pink for locations) in orientation and location trials. (D) Use of the words ‘top’ (green for orientations, dark pink for locations) and ‘bottom’ (blue for orientations, light pink for locations) in orientation and location trials. (E) Use of the word ‘left’ in orientation and location trials. (F) Use of the word ‘right’ in orientation and location trials. (G) Correlations between recall error for orientations (green bars) and locations (pink bars) and the frequency of use of spatial words across stimuli. Upwards directed bars indicate positive correlations and downward directed bars indicate negative correlations.

Spatial language is unique since spatial terms describe unevenly distributed and relatively vague concepts when compared, for example, to the evenly distributed hours on a clock or cardinal directions on a compass. Different spatial terms divide the circular space differently (e.g. ‘left’ and ‘right’ versus ‘diagonal’, ‘horizontal’ and ‘vertical’; see Figure 3A–F). We evaluated whether the patterns of labeling behavior across the stimulus space predicted the variation in recall error during the delayed estimation task (i.e. class 1 cardinal biases, Figure 3G).

The use of ‘diagonal’ correlates positively with the recall error for orientations (*r* = .54, *p* = .006, see Figure 3A) and for locations (*r* = .5, *p* = .012, see Figure 3A). This indicates that the more frequently a person uses the term ‘diagonal’, the more imprecise their response. On the other hand, the use of the words ‘vertical’ and ‘horizontal’ combined inversely correlates with recall error (orientations: *r* = –.54, *p* = .006, locations: *r* = –.42, *p* = .039, see Figure 3B). ‘Left’ and ‘right’ are the most commonly employed words for both stimuli, and when grouped together their usages correlate with recall error (orientations: *r* = .82, *p* < .001, locations: *r* = .89, *p* > .001, see Figure 3E–F). Recall error for orientations (but not locations) also correlates with ‘top’ (*r* = .5, *p =* .012) and ‘bottom’ (*r* = .43, *p =* .037), and location stimuli recall error inversely correlates with ‘up’ (*r* = –.41, *p* = .04, see Figure 3C–D). No correlation with either stimulus type was found for the word ‘down’. When grouping the usage of these four words together, we observed a negative correlation with the location recall error pattern (*r* = –.4, *p* = .044). Approximation words – ‘slightly’, ‘almost’, ‘near’, ‘just’ – did not correlate with absolute error in the delayed estimation tasks for neither orientation nor location stimuli (all *p* > .1; see Figure 3G).

Thus, spatial words that are symmetrically used in all spatial quadrants of the visual field are highly predictive of recall error and therefore of class 1 cardinal biases. Other spatial words used less symmetrically also show some correlations but tend to be less predictive overall. Notably, when paired with their opposite field counterparts they show more robust correlations as well.

### 3.4 Approximation terms indicate susceptibility to bias

**Figure 4:**
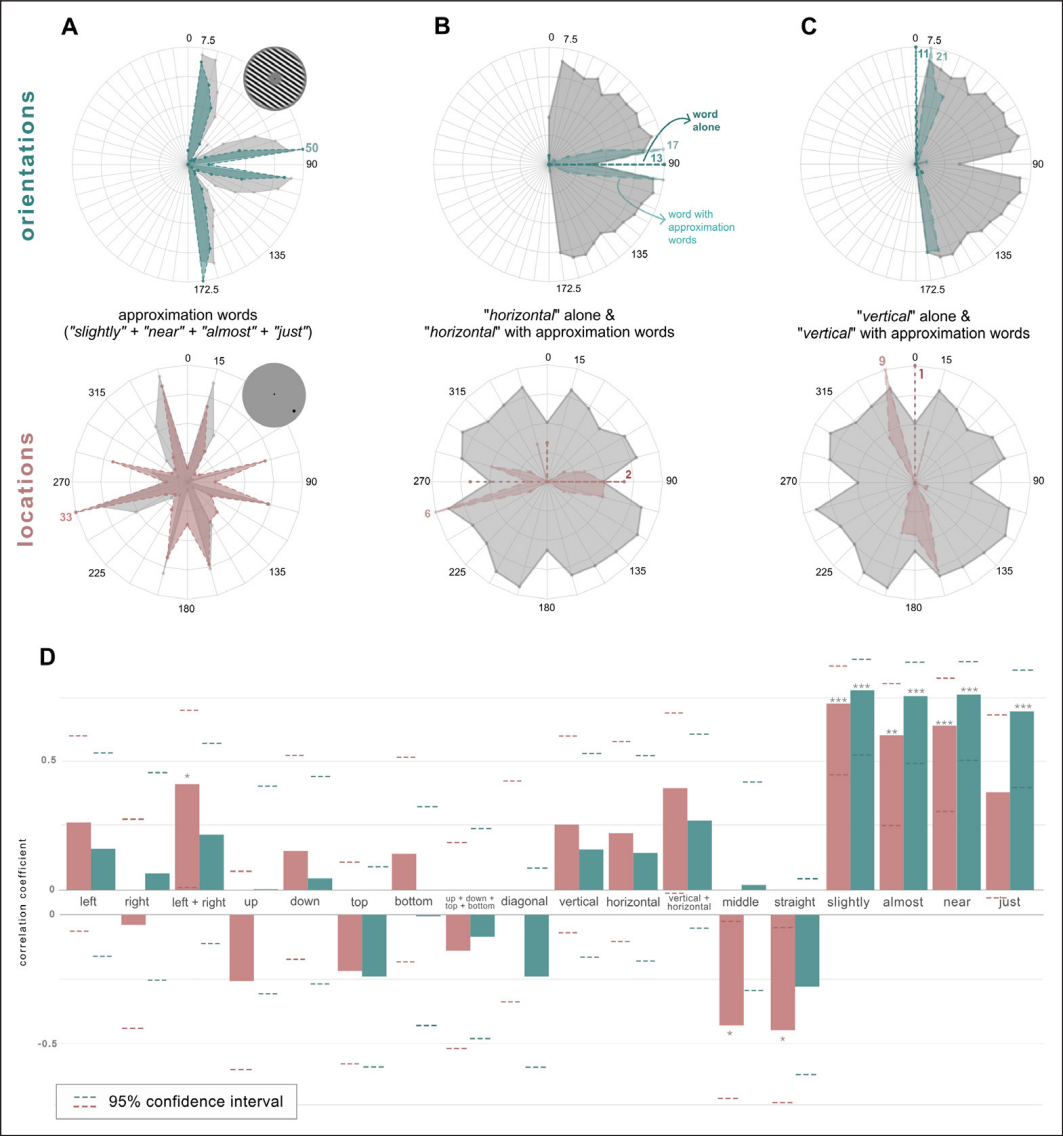
Approximation Words Usage During the Naming Task. *Note.* Distribution of usage of approximation words across the stimulus space and their correlation to recall bias in delayed estimation task. (A) Frequency of approximation words use in orientation (upper figure) and location (lower figure) trials during the naming task. The shaded green area corresponds to the amount of times an approximation word was used for a given stimulus, and the number with matching color indicates (in the stimulus in which it was used more frequently) the number of participants who employed it to describe that specific stimulus. The gray area indicates the average recall bias for each stimulus. (B) Usage of the word ‘horizontal’ with and without approximation words in orientation (upper figure) and location (lower figure) trials during the naming task. The green or pink area indicates word use with approximation words, and the dashed line corresponds to use of the word on its own. The gray area indicates the average absolute recall error for each stimulus. (C) Usage of the word ‘vertical’ with and without approximation words in orientation (upper figure) and location (lower figure) trials during the naming task. The green or pink area indicates word use with approximation words, and the dashed line corresponds to use of the word on its own. The gray area indicates the average absolute recall error for each stimulus. (D) Correlations between recall bias for orientations (green bars) and locations (pink bars) and the frequency of use of spatial words across stimuli. Upwards directed bars indicate positive correlations and downward directed bars indicate negative correlations.

We tested whether the use of approximation words predicted larger repulsion biases (class 2 cardinal biases, see Figure 4) and found significant correlations for both orientation and locations repulsion biases with almost all words: ‘slightly’ (orientations: *r* = .77, *p* < .001, locations: *r* = .72, *p* < .001), ‘near’ (orientations: *r* = .75, *p* < .001, locations: *r* = .63, *p* < .001), ‘almost’ (orientations: *r* = .75, *p* < .001, locations: *r* = .59, *p =* .002) and ‘just’ (orientations: *r* = .69, *p* < .001), with the exception of ‘just’ in locations (*r* = .38, *p =* .067) (see Figure 4). Recall biases for location stimuli inversely correlate with the use of ‘straight’ (*r* = –.4, *p* = .03) and ‘middle’ (*r* = –.4, *p* = .04). No other words were found to have a significant correlation with recall bias (all *p* > .05).

### 3.5 Shared and unique strategies for different features

**Figure 5:**
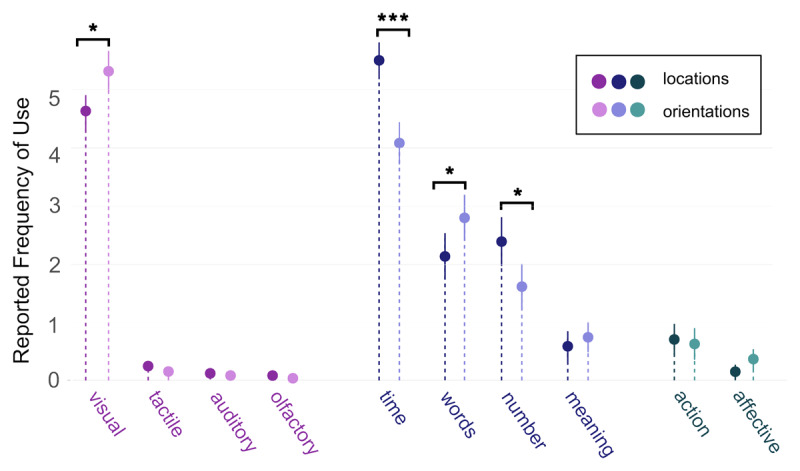
Self-reported Use of Encoding Strategies (in Post-experimental Questionnaire). *Note.* Participants’ average rating in response to post-experimental questionnaire about memorization strategies of different modalities. Participants were asked if they used any of the strategies in the x axis in the post-experimental questionnaire (e.g. for ‘action’, the question was ‘I memorized the orientations/location during the delay period through a related action’), and were asked to rate how much they believed they relied on it during the task for orientation stimuli, and for location stimuli (see Appendix A).

After the experiment, participants responded to a series of questions about the strategies they employed to perform the delayed estimation task (see Appendix A, Figure 5). This included questions about encoding strategies beyond verbalization. Overall, orientations and locations share a common pattern of encoding strategies but subjects’ responses to the questionnaire indicate differences between the two stimulus types for the following strategies: visual (Z = –2.46, *p* = .013), time (Z = –4.53, *p* < .001), words (Z = –2.02, *p* = .043), and number (Z = –2.47, *p* = .013, but see Appendix B). Locations are rated higher for the first two strategies and orientations for the other two.

## 4. Discussion

### 4.1 Subjects prefer high-specificity verbalization during visual memory

In the present study, we set out to investigate verbal strategies during visuo-spatial working memory. We found that our participant sample, comprised of 108 english speakers, of which 79 are monolingual native English-speaking subjects from a vast variety of countries (United Kingdom, New Zealand, Australia, Ireland, Sweden, Zimbabwe, India, United States, Canada, Bermuda), show a robust conceptual mapping between stimuli and certain labels. Spatial language was the most used labeling strategy and its use both as a broad category and for individual words predicted recall errors reliably. The clock strategy was also frequently employed. This consistent use of groups of words among participants might be partially due to the stimulus simplicity, which has been shown to elicit less labeling possibilities than complex abstract stimuli (Brown et al., 2006). Assessing the same measures with these more complex stimuli could provide valuable insights. The use of approximation words reliably predicted recall biases from the delayed estimation task. Participants reported verbal strategies and visual analogies as the most helpful resources for completing the task in a post-experimental questionnaire.

It is reasonable to believe that the clock and spatial language strategies are popular among participants since they both provide a wide set of diverse terms, allowing for labeling specificity (Souza et al., 2021). In the clock strategy, a single word can confer high specificity based on a visual analogy that is well-known by most people. In contrast, spatial terms are frequently grouped together, taking advantage of often overlapping yet different divisions of the space to provide labeling specificity (see 3.2 ‘Subjects mostly use spatial and clock-analogous terms’; Figures 3 and 4). Notably, variation in labeling specificity across subjects or stimuli is not predictive of performance, but it is unclear whether the number of responses in the naming task are a sufficiently reliable indicator of labeling specificity.

### 4.2 Does verbalization explain cardinal biases?

Our results provide a possible explanation of cardinal biases, both type I, visual, and type II, cognitive (Appelle, 1972; Balikou et al., 2015; Essock, 1980). The overlap between the usage of words with the areas of higher error, higher accuracy or recall biases shows the predictive power of these labels. Similar relations between verbal labeling and categorical biases for other stimulus types have been described for colors (Bae et al., 2015; Souza et al., 2021; Yan et al., 2023), shapes (Gauthier et al., 2003; Souza et al., 2021) and objects (Gauthier et al., 2003; Lupyan, 2008; Lupyan et al., 2007). Verbalizing the general category (e.g. ‘car’, ‘face’, ‘house’) of complex visual stimuli seems to pose no additional processing time compared to object detection alone (Grill-Spector & Kanwisher, 2005) suggesting that verbalization generates no or very little cost during encoding. It is worth noting that the verbal labeling and recall error measures in our study were obtained in different tasks, and therefore we cannot be sure participants were attributing verbal labels during the delayed estimation task. However, performance in silence conditions that provide ample time for labeling is similar to performance in overt labeling conditions, and both are better than under verbal suppression (Forsberg et al., 2020; Overkott et al., 2023; Souza & Skóra, 2017). Hence, the long presentation time during this task is likely to induce verbalization. Additionally, given that no differences in performance were found between the participants who received the prompt to ‘think of words to describe the stimulus’ prior to the delayed estimation task and those who did not, it is possible verbal labeling occurs regardless.

On the neural level, evidence for categorical cortical representations of orientations and locations has been reported for artificial categories in the occipitoparietal cortex (Ester et al., 2020). It has remained unclear, however, what role verbal labeling played for these newly learned stimulus classes. For color stimuli, categorical memory representations have been identified in the extrastriate visual cortex for common color categories (e.g. ‘red’, ‘green’, ‘blue’) even when subjects received no instruction to categorize the stimuli (Yan et al., 2023). These categorical representations were identified using encoding models generated using labeling data, suggesting a link between these categorical representations, categorical biases, and verbalization.

It is clear, however, that categorization and semantic processing can occur without human-like verbalization. Non-human primates can distinguish behaviorally between classes of stimuli, and neurons across a number of regions can show category-selective responses (Freedman et al., 2003; Sigala & Logothetis, 2002). Patients with naming deficits are able to categorize colors (Siuda-Krzywicka et al., 2019). For low level stimuli such as orientations, neuronal selectivity prioritizing cardinal stimuli has been found in the primary visual cortex of mice (Roth et al., 2012), cats (Wang et al., 2003) and macaques (Fang et al., 2022). This line of research argues that cardinal biases might occur due to biologically embedded priors, and that their presence in cortical representation and recall is a consequence of features of natural images (A-Izzeddin et al., 2023; Girshick et al., 2011; Hansen & Essock, 2004) or to general environment (Coppola et al., 1998).

While biological priors for cardinal biases might be present in humans and other mammals, this does not mean verbal processes play no part in categorization in humans. Previous research has observed benefits for labeling of low-level stimuli prone to categorical biases with both their common labels and with artificial, ‘meaningless’ labels (Gauthier et al., 2003; Lupyan et al., 2007; Overkott & Souza, 2023; Souza et al., 2021). Thus, verbalization, non-verbal categorization, and environmental priors might contribute to cardinal biases.

### 4.3 Uncertainty and specificity in verbal labeling

While labeling can be beneficial in working memory tasks and help us remember stimuli more accurately, it can also induce biases. Less prototypical items of a category usually exhibit more error upon recall (Bae et al., 2015; Souza et al., 2021). Verbal recall of previously seen visual stimuli can impair subsequent visual recall (Schooler & Engstler-Schooler, 1990), and can be attenuated through the re-introduction of visual cues (Brandimonte et al., 1997). Naming the broad category a stimulus belongs to can lead to more confusion in a recognition task where plenty more stimuli belonging to the same category are also present (Lupyan, 2008), and to less precise memory in delayed estimation tasks (Souza et al., 2021).

In our data, several stimuli were labeled based not on their own features but on their relation to other stimuli, with the use of approximation words, e.g. ‘almost horizontal’ or ‘slightly off vertical’. These stimuli elicited the highest recall biases. The use of approximation words seems to indicate uncertainty in the labeling of these stimuli, once again attesting for the relation between categorical ambiguity and poorer working memory performance. This result mimics the correlation between verbal description length and visual stimulus complexity in prior work (Glanzer & Clark, 1964).

While using more words to describe a single stimulus might indicate uncertainty, having more diverse labels to choose from seems to provide and advantage (Overkott & Souza, 2023; Souza et al., 2021). Souza et al (2021) compared a 2-word labeling condition in which participants could only label the memoranda with two terms against a 4-word labeling condition in which four terms had to be used. Working memory recall was better in the 4-word condition, likely due to the increased labeling specificity provided by more naming options. Here, we did not find a labeling specificity benefit, but the current study is less suited for this as we did not vary or measure labeling specificity on a trial-wise level. It could also be due to the use of visuo-spatial and not color stimuli in the current study. An investigation of free labeling for both stimulus types would be a great resource to try and disambiguate potential labeling behavior differences between simple yet distinct low-level stimuli.

### 4.4 The role of verbal strategies in visuo-spatial working memory

Verbal labeling is frequently present during visuo-spatial working memory. Whether this is a main driver of categorical biases or part of a multi-causal chain, the close relation between the use of verbal strategies and categorization is hard to deny. This interaction is evident in a number of stimuli: in complex high-level items (Gauthier et al., 2003; Lupyan et al., 2007), in easily verbalized low-level stimuli such as colors (Bae et al., 2015; Souza et al., 2021; Yan et al., 2023) and in stimuli where the labels are not so intuitive, like orientations, spatial frequency, locations and shapes (Overkott & Souza, 2023; Souza et al., 2021). Even in stimuli for which the names are words we have never used before, there is an advantage for prototypical category members (Overkott & Souza, 2023; Souza et al., 2021). While verbalization seems to have a prominent role in viso-spatial working memory, other strategies might occur concurrently (see Figure 5, Brown & Wesley, 2013) and, even within verbalization, several verbal recoding strategies can take place (Gonthier, 2021). There is reason to believe that individual items are concurrently represented in multiple cortical regions using several different representational formats across the cortex (Christophel et al., 2017; Ester et al., 2015; Salazar et al., 2012). Verbal and categorical representations can be found in language-related regions (Koenigs et al., 2011; Yan et al., 2021; Yue et al., 2019) and even in sensory cortex (Popham et al., 2021; Yan et al., 2023). Recurrent activation between verbal-categorical and sensory representations might be essential for maintaining persistent activity in either region (Iamshchinina et al., 2021). These cross-cortical reverberations might explain why categorical biases are stronger during working memory compared to perception (Bae et al., 2015; Yan et al., 2023) as ongoing regional interaction might push the overall representation towards a more categorical code. Verbalization might be more pronounced when higher loads lead to stronger interference within visual cortex (Chopurian et al., 2024; Zhou et al., 2022) and this might help increase working memory performance (Souza & Skóra, 2017). Thus, there is reason to believe that verbalization is an essential part of human working memory abilities even for low-level visual memoranda. Understanding this part of visual working memory is therefore a prerequisite for understanding the whole.

## Data Accessibility Statement

Experimental data can be found at: https://osf.io/n27jd/.

## Appendix A

### Post-Experimental Encoding Strategy Questionnaire

This appendix consists of the questionnaire on encoding strategies participants were presented with after concluding the experiment. There was a block of questions for each stimulus type, which included rating scale questions and an open ended question. The order of the question blocks was randomized, and so were the statements within each rating question.

In the questionnaire below, [stimulus type] is a placeholder for “orientations (i.e. stripped stimuli)” and “spatial locations (i.e. dots)”, in each of the question blocks.

### Question 1

I memorized the [stimulus type] during the delay period…

- by using words to describe them
- by giving them some name, code or number
- through a related smell or taste
- through a related action
- by thinking of another image (e.g. a photograph, a logo, etc)
- by moving my hands
- through an associated emotion
- through an associated touch
- by what they might mean
- by how they might sound
- by how they looked
- through an associated time i.e. pointer position on a clock

### Question 2

I memorized the [stimulus type] during the delay period … in a way that was not described before (if yes, please explain).

## Appendix B

### Additional Analysis Concerning Labeling Specificity and Stimulus Similarity

The overall pattern indicates a shared set of verbalization strategies for orientations and locations. The use of words that describe a symmetric property (i.e. ‘diagonal’, ‘horizontal’, ‘vertical’) and approximation words is correlated across the two features (diagonal *r* = .93; horizontal *r* = .77; vertical *r* = .93; slightly *r* = .75; near *r* = .79; almost *r* = .8; just *r* = .72; all *p* < .05). Reversely, spatial words that describe an asymmetric property (e.g. ‘up’, ‘right’) are almost always not correlated (down *r* = .21; right *r* = .46; left *r* = –.15; all *p* > .1; with the exception of up *r* = .61, *p* = .032).

We also tested if there was a benefit of using more specific labeling strategies in our study. We addressed this question by using correlations between recall error and number of spatial words or strategies using across subjects and stimuli. We also tested whether number of unique labels per subject correlated with recall error (see methods for details). We found no significant correlation between these measures of labeling specificity and overall performance across participants (all *p* > .05).
